# Supplementary figures and images for: Novel role of SARM1 mediated axonal degeneration in the pathogenesis of rabies
Source: PLoS Pathog. 2020 Feb 18;16(2):e1008343. doi: 10.1371/journal.ppat.1008343 (PMC7048299; doi:10.1371/journal.ppat.1008343)

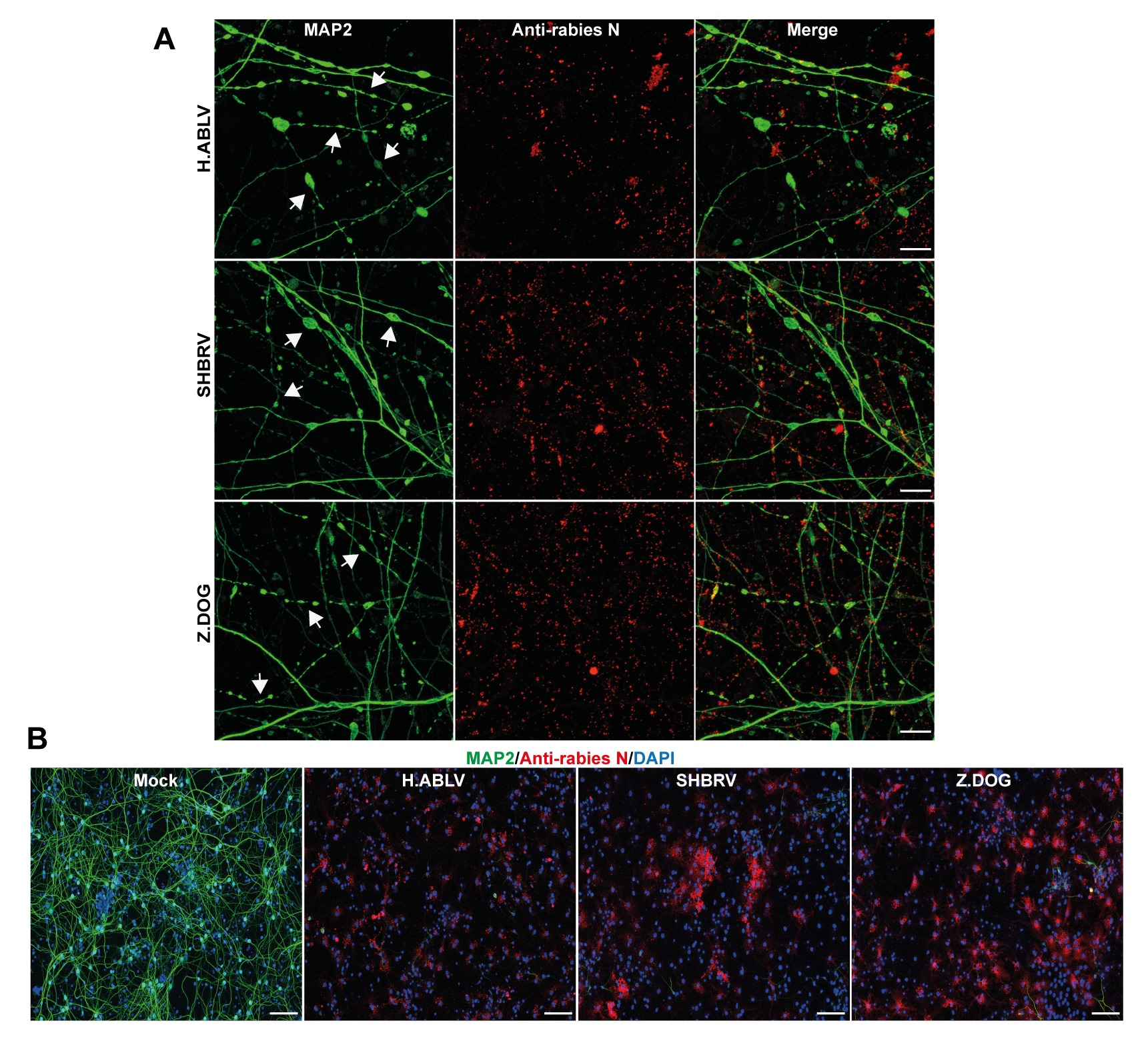

Supplement: S1 Fig — (A) Rabies induced blebbing of MAP2-positive neurites in cortical neurons at 16 hours post infection (high magnification). Representative confocal images of mouse primary cortical neurons infected with rabies strains (H.ABLV, SHBRV and Z.Dog), showing focal swellings in the MAP2-positive neurites (white arrows) at 16 hours post infection. Neurons are stained with anti-MAP2 antibody (green) and anti-rabies nucleoprotein antibody (Red). Scale bar 10 μM. (B) Rabies induced loss of MAP2-positive neurites in cortical neurons at 24 hours post infection (low magnification). Representative stitched tile images of mouse cortical neurons infected with rabies strains (H.ABLV, SHBRV and Z.Dog) showing loss of MAP2 staining at 24 hours post infection, but not in mock infected neurons. Scale bar 100 μM. Neurons are stained with anti-MAP2 antibody (green), anti-rabies nucleoprotein antibody (red) and DAPI (blue). Merged images of all three channels are shown. (TIF) [file ppat.1008343.s001.tif]

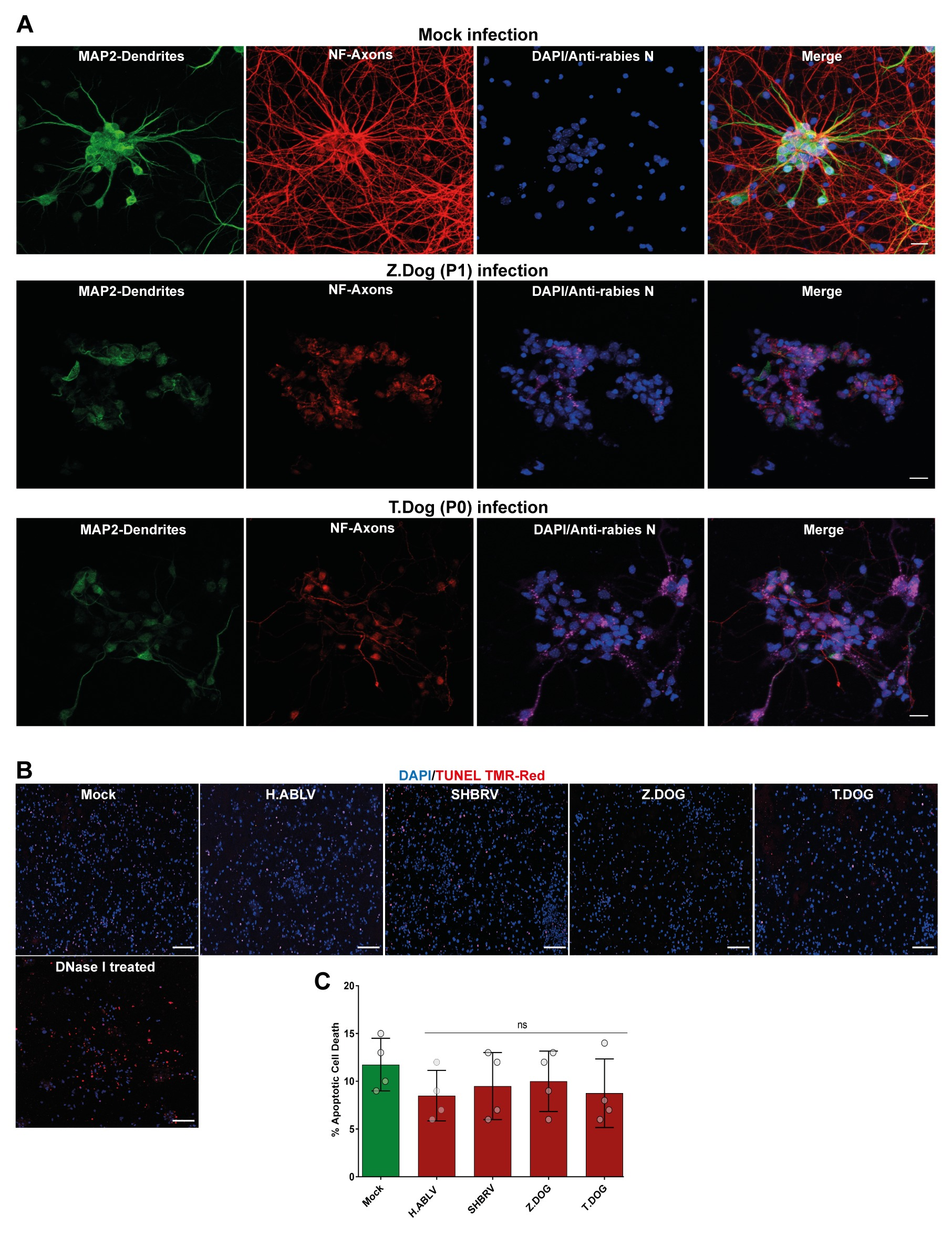

Supplement: S2 Fig — (A) Representative confocal images of mouse primary cortical neurons infected with parental stocks of rabies strains (Z.Dog P1 and T.Dog P0). High magnification images of mock infected neurons show co-staining of shorter dendrites with MAP2 antibody (green) and longer axons with neurofilament antibody (red) in the same neurons. Infection with Z.Dog P1 and T.Dog P0 strains for 24 hours, show significant loss of filamentous dendritic and axonal staining in neurons. Rabies infection is identified by staining with anti-rabies nucleoprotein antibody (magenta) and nuclei are stained in blue. Scale bar 20 μM. (B) Examination of induction of apoptosis in primary cortical neurons infected with rabies for 24 hours. Representative stitched tile images of mouse cortical neurons infected with rabies strains and stained with TUNEL to detect apoptosis. As a positive control, neurons were treated with DNase I to induce apoptotic DNA fragmentation. Images show neurons stained with DAPI (blue-nuclei) and TUNEL (red-apoptotic neurons). Scale bar 100 μM. (C) Quantification of apoptotic neurons in rabies infected and mock infected cultures. Percentage of apoptotic neurons was calculated from experiments shown in S2B Fig using ImageJ. TUNEL positive neurons were identified as apoptotic and the percentage of apoptosis was calculated from the total number of DAPI stained nuclei. At least 500 neurons are quantified per sample. Data was found to be not significant versus mock infected neurons, n = 4. Images shown are maximum intensity projections of Z-stacks. (TIF) [file ppat.1008343.s002.tif]

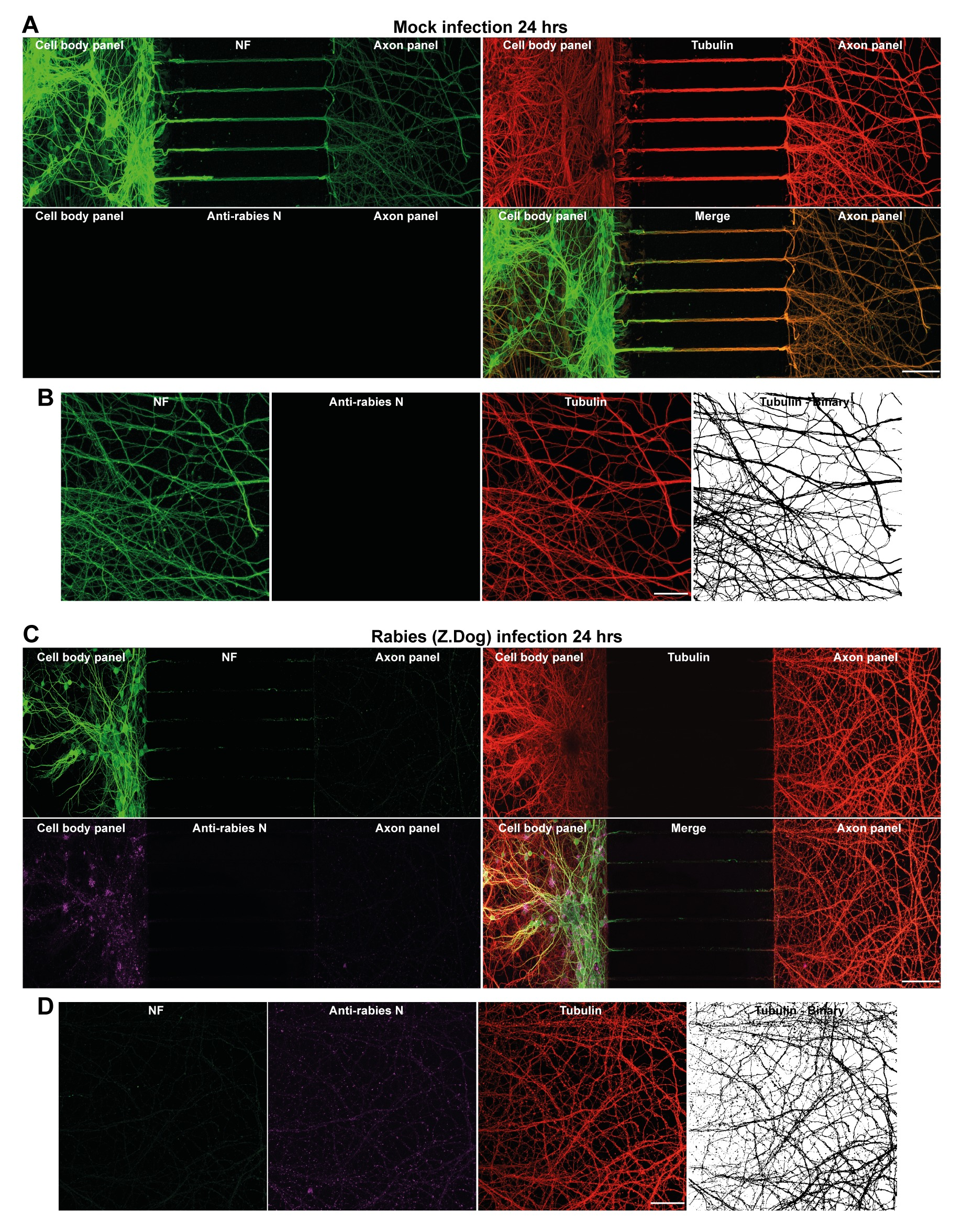

Supplement: S3 Fig — (A) Immunostaining of mock infected primary cortical neurons cultured in microfluidic chamber. Stitched tile confocal image showing the microfluidic chamber seeded with cortical neurons on the cell body panel. As the neurons grow, axons extend through the micro-channels into the axon panel. The neurons were mock infected on the axon panel for 24 hours and a higher volume of culture media was maintained on the cell body panel to maintain the flow of media from cell body to axon panel and avoid random diffusion of inoculum from the axon panel. Neurons are stained with anti-neurofilament antibody (NF, green), anti α-tubulin (red) and anti-rabies nucleoprotein antibody (magenta). Scale bar 100 μM. (B) Higher magnification images of the axon panel from the mock infected neurons shown in S3A Fig. Images show intact neurofilament staining and filamentous tubulin stained axons. Scale bar 50 μM. (C) Immunostaining of primary cortical neurons cultured in microfluidic chamber and infected with Z.Dog rabies virus for 24 hours in the axon panel. Stitched tile confocal image show loss of neurofilament staining in the axons of neurons infected with rabies for 24 hours, while neurofilament staining can still be observed in the cell bodies and the proximal axons in the cell body panel. Similarly, tubulin staining in the axon panel show granular disintegration of distal axons. Rabies infection is identified by positive staining with anti-rabies nucleoprotein antibody. Scale bar 100 μM. (D) Higher magnification images of axon panel from the Z.Dog rabies infected neurons shown in S3C Fig. Images show axonal degeneration as observed by the loss of neurofilament staining and granular disintegration of tubulin stained axons. Scale bar 50 μM. Images shown are maximum intensity projections of Z-stacks. (TIF) [file ppat.1008343.s003.tif]

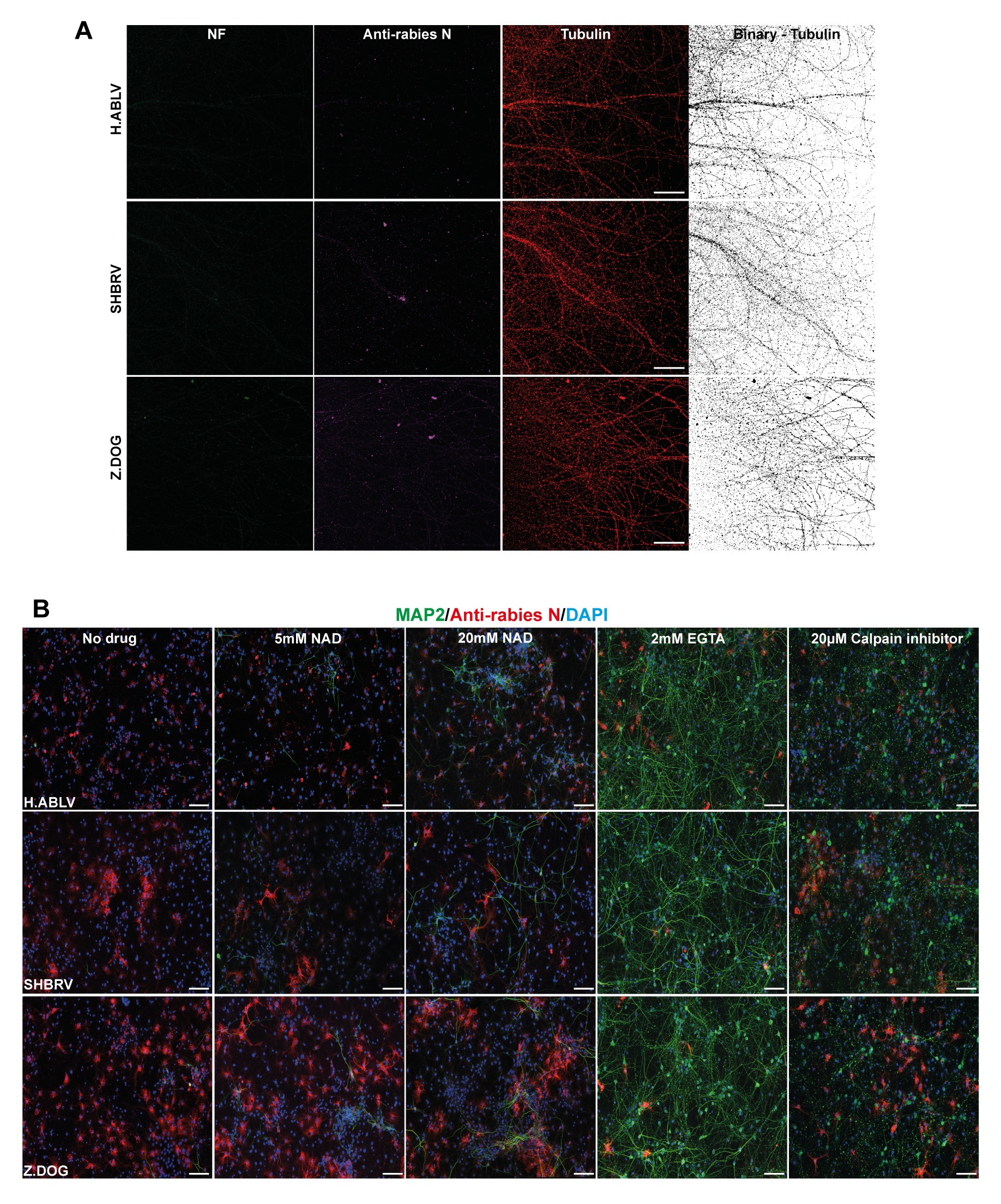

Supplement: S4 Fig — (A) Rabies induced axonal degeneration in primary DRG neurons cultured in microfluidic chambers. Mouse DRG neurons were cultured in microfluidic devices to separate the cell body and axons. The axon panels were then infected with different street strain rabies virus (H.ABLV, SHBRV and Z.Dog) for 24 hours. Figure shows stitched tile images of axon panel infected with rabies virus. The axons were stained with pan axonal neurofilament antibody (green), anti-rabies nucleoprotein antibody (magenta) and anti α-tubulin (red). Scale bar 100 μM. Axonal degeneration is observed by the loss of neurofilament staining and granular disintegration of tubulin stained axons. Images shown are maximum intensity projections of Z-stacks. (B) Pharmacological inhibition of neurite degeneration in rabies infection. Effect of extracellular NAD addition and calpain inhibition on rabies induced loss of MAP2-positive neurites. Stitched tile images of mouse cortical neurons infected with H.ABLV, SHBRV and Z.Dog rabies for 24 hours and treated with NAD, EGTA or Calpain inhibitor III. Scale bar 100 μM. Neurons are stained with anti-MAP2 antibody (green), DAPI (blue) and anti-rabies nucleoprotein antibody (red). All three channels are merged together, which show reduced loss of MAP2-positive neurites in infected neurons treated with NAD, EGTA or calpain inhibitor III compared to no drug treatment. Images shown are maximum intensity projections of Z-stacks. (TIF) [file ppat.1008343.s004.tif]

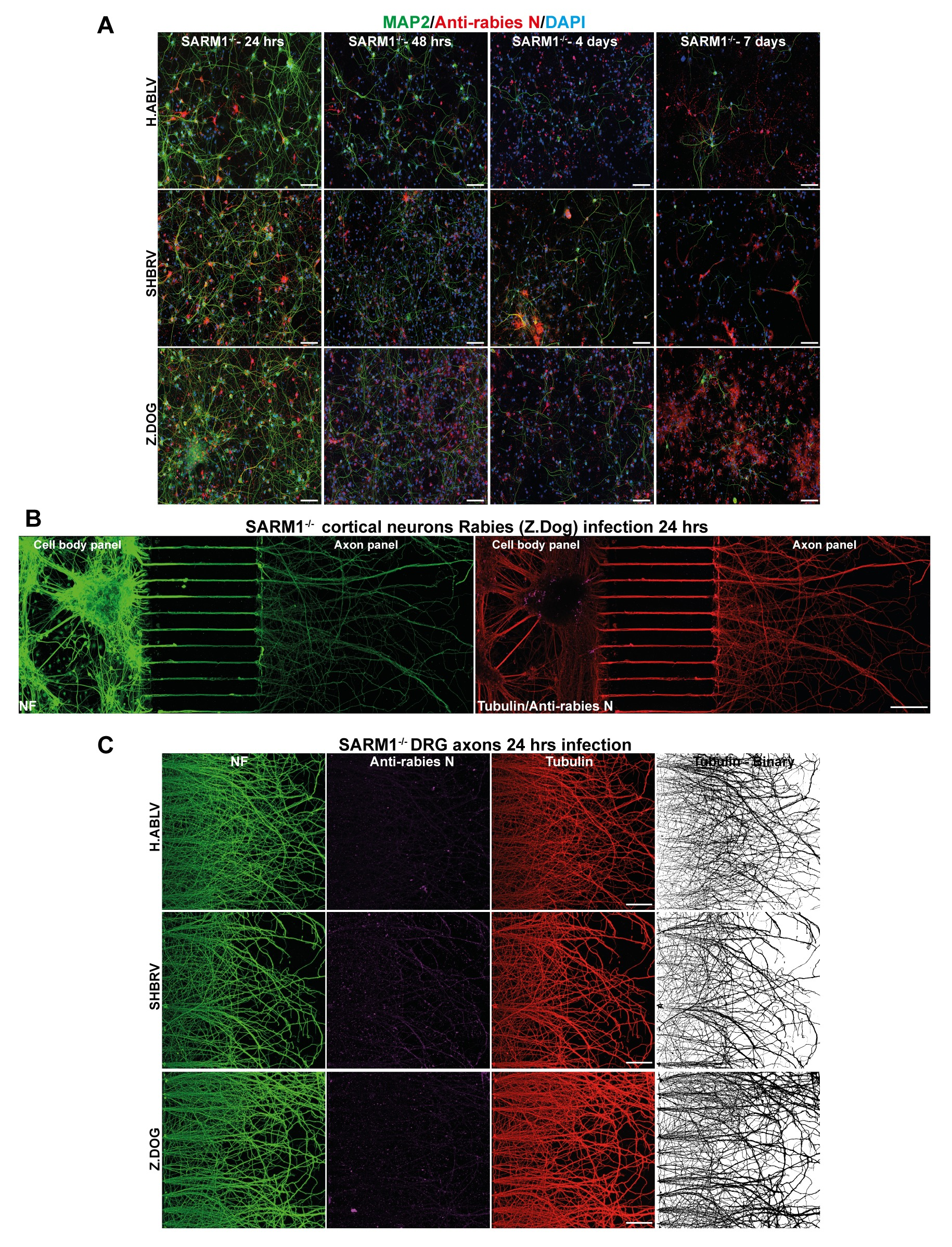

Supplement: S5 Fig — (A) Time course analysis of neurite degeneration in SARM1 knockout neurons infected with rabies. Representative images of mouse cortical neurons from SARM1 knockout (SARM1-/-) mice infected with rabies virus (H.ABLV, SHBRV and Z.DOG) for 24 hours to 7 days. Images show intact MAP2-positive neurites in rabies infected SARM1-/- neurons at 24 hours, but a gradual loss of MAP2-positive neurites extending up to 7 days. Neurons are stained with anti-MAP2 antibody (green), anti-rabies nucleoprotein antibody (red) and DAPI (blue). Scale bar 100 μM. (B) Immunostaining of SARM1-/- cortical neurons cultured in microfluidic chamber and infected on the axonal panel with Z.Dog rabies virus for 24 hours. Stitched tile confocal image shows SARM1-/- neurons infected on the axon panel for 24 hours. Neurons are stained with anti-neurofilament antibody (NF, green), anti α-tubulin (red) and anti-rabies nucleoprotein antibody (magenta). Scale bar 100 μM. Image shows lack of axonal degeneration in SARM1-/- neurons infected specifically at the axons with rabies virus. (C) Inhibition of rabies induced axonal degeneration in SARM1-/- DRG neurons cultured in microfluidic chambers. Primary DRG neurons from WT and SARM1-/- mice cultured in microfluidic devices to separate the cell body and axons. The axon panels were then infected with different street strains of rabies virus (H.ABLV, SHBRV and Z.DOG) for 24 hours. Figure shows stitched tile images of axon panel of SARM1-/- neurons infected with rabies. The axons were stained with anti-neurofilament antibody (green), anti α-tubulin (red) and anti-rabies nucleoprotein antibody (magenta). Scale bar 100 μM. Images shown are maximum intensity projections of Z-stacks. (TIF) [file ppat.1008343.s005.tif]
