# Supplementary material for: Novel role of SARM1 mediated axonal degeneration in the pathogenesis of rabies
Source: PLoS Pathog. 2020 Feb 18;16(2):e1008343. doi: 10.1371/journal.ppat.1008343 (PMC7048299; doi:10.1371/journal.ppat.1008343)
Supplement: Attachment — Submitted filename: Revision cover letter with responses.pdf [file ppat.1008343.s008.pdf]

CSIRO
Australian Animal Health Laboratory
5 Portarlington Road, Geelong, Victoria 3220 Australia

csiro.au | ABN 41 687 119 230

20th December 2019

Dear Editor,

We thank the reviewers for a comprehensive review of our manuscript. We are pleased to note that all three reviewers found this to be an excellent study, describing an important neurobiological mechanism in rabies. We have now fully reviewed the concerns raised by reviewers and we are confident that the revised manuscripts along with the responses provided below addresses all the concerns raised by the reviewers. We request you to reconsider this revised manuscript for publication in *PLOS Pathogens*.

Comments from editorial decision

Reviewer #1 raised an important point that unequivocal discrimination between axons and dendrites is critical for the authors' underlying mechanistic interpretation; as is stands, the MAP2 staining alone is insufficient to make this discrimination for the reasons stated by the reviewer.

Response: *We agree with reviewer#1 that MAP2 staining alone cannot identify axons, as MAP2 is specific to dendrites [1, 2]. For this reason, in the study, we have specifically stained for axons using pan-axonal neurofilament antibody/SMI-312 (BioLegend, Cat#837904), in addition to MAP2. This data is shown in figures 1F, 1H, 1D, 3C, 3E, 3G, suppl. Fig 2A, suppl. Fig 3, suppl. Fig 4A, suppl. Fig 5B, suppl. Fig 5C (revised figure#). Neurofilament is highly abundant in the axons [3, 4]. The antibody (SMI-312) used in this study is a combination of monoclonal antibodies targeted against different neurofilament epitopes, which was shown to identify mature axons [5, 6, 7]. As demonstrated in many previous studies, this SMI-312 antibody along with MAP2 antibody could be specifically used to distinguish between axons and dendrites of neurons [8, 9, 10, 11]. In addition, we have used a microfluidic chamber system to isolate axons of neurons (suppl. Fig 3, revised figure#). These chambers with 450 µM long microchannels have been previously shown to effectively separate the axonal and the cell body-dendrite culture compartments [12]. We have performed our analysis specifically on the axons separated using this microfluidic chamber by staining for axonal neurofilament, ensuring an explicit identification of axons. While we only show the loss of MAP2-stained dendrites in Fig 4, in the lead up to this data, we have clearly demonstrated that both MAP2-positive and neurofilament-positive axons degenerate in response to rabies infection. In Figure 4, we have chosen MAP2 staining for representation and comparison of SARM1-mediated pathology in the rabies-infected panels of WT and SARM1 knockout neurons cultured in microfluidic chambers. MAP2 antibody is a superior stain for this purpose, being able to identify cell bodies and associated dendrites of neurons in the specific panels. However, the mechanistic interpretation of the data presented in Figure 4, is led by a clear demonstration of degeneration of both axons and dendrites in Figures 1 and 3 (including supplementary figures). We do acknowledge that axonal degeneration is not specifically shown in Figure 4 with only MAP2 staining, and hence we have now removed a direct reference to axonal degeneration in this Figure (page 9 line 224 and line 231).*

To further highlight the distinction between axons and dendrites we have now repeated infections in primary cortical neurons and co-stained using MAP2 and neurofilament antibodies to enable parallel identification of dendrites and axons. This new figure (suppl. Fig 2A) clearly shows the distinction of shorter dendrites closer to the cell body by MAP2 staining and much longer axons identified by neurofilament staining on the same neurons. This image is shown below for reference. In addition, we show the

degeneration of both MAP2-positive dendrites and neurofilament-positive axons upon infection with rabies in the same neurons (suppl. Fig 2A, revised figure#), which is consistent with all other data described in the original manuscript.

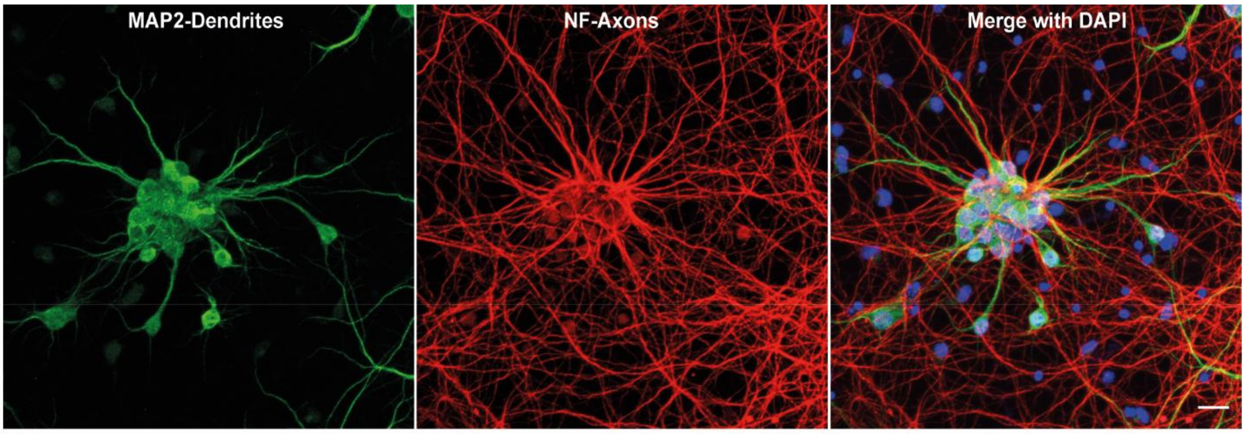

Reviewer #2 had some concerns with the utilized RABV strain - you should consider confirming some of your results with another RABV strain to ensure it's not specific to the one used RABV strain. Many of Reviewer #2's other concerns could also be addressed by further explication or toning down the conclusions.

Response: *In this study, we have studied the pathogenesis of not one but four different strains of lyssavirus, which are field isolates from infected dog, bat or horse. While quantification data from experiments using all the rabies strains are shown in the main figures, representative images from three additional rabies strain infections are shown in supplementary images. We agree with reviewer#2 that subjecting these rabies strains to even few rounds of passaging in suckling mouse brain and Neuro-2a cells may influence the pathogenicity or generate viral subpopulation. While we would have liked to use stock virus from original brains, this was not possible due to limited quantities, and therefore it was necessary to passage the viruses to build up our stocks. To address this concern, we have repeated the experiment with lowest-pass parental stocks of two of our rabies virus strains. These stocks are either directly prepared from the brain homogenates of infected dog (Thai Dog P0) or from the sucking mouse brain after first passaging of viral stocks (Zimbabwe dog P1). In this experiment, we show that these original viral stocks induce axonal and dendrite degeneration in primary neurons similar to the viral stocks which are minimally passaged and used in the main parts of this study. Hence, we now show that the pathogenic mechanism identified in this study is not influenced by minimal passaging of viral strains. This new data is now added to the revised manuscript (suppl. Fig 2A).*

Comments from reviewers

Reviewer #1: In this paper, Sundaramoorthy et al., show that cultures of primary cortical and peripheral neurons infected with different lyssavirus strains (rabies virus, RABV) lose structural proteins and demonstrate neurite degeneration. This is a striking phenotype resulting in near complete loss of neurite structures by 24 hours after infection, supported by MAP2, NF and tubulin antibody staining. They also show that SARM1 knockout mouse neurons, when infected with RABV, show delayed neurite degeneration with subsequent enhanced transneuronal RABV spread in cortical neurons cultured in connected microfluidic chambers. This is an important finding hinting at an intrinsic neuronal defense mechanism against viral spread possibly leading to RABV related neuropathologies. Although, the paper was written clearly and results were supported by clean images and quantitation, some critical data was missing.
One of the critical points missing is the discrimination between axons and dendrites. The authors claim in many experiments that the neurites referred to are axons (particularly in the last figure), however, only MAP2 staining is shown. MAP is abundant in somatodendritic regions, but not axons. This point is

important because RABV is known to spread retrogradely between connected neurons after entering axons of motor neurons from neuromuscular junctions. That fact means that the endocytosed particles are transported in the axons, the genomes are released and replicate in the neuronal soma, and progeny spread from dendrites to connected axons. If the infected cell axon degenerates, it will not reduce the somatodendritic spread in the infected neuron. If all neurites (both axons and dendrites) are degenerating due to a loss of MAP2, then SARM1 mediated axon degeneration is not the predominant mechanism blocking transneuronal viral spread. As an alternative hypothesis, recall that Sarm1 was originally identified as a negative regulator of TLR3 and TLR4 pathways in innate immunity (Carty et al., 2006, Nat. Immunol.). This negative regulation might be responsible for the virus yield phenotype in Sarm1 knock out neurons. This alternative hypothesis is important particularly for interpreting the last figure (F4). It appears that there is more viral antigen staining in the inoculated chamber of SARM1 knock out neurons. However, in figure 3, it appears that there is less viral antibody staining 24 hours after infection, but staining catches up after 4 days. Virus yields should be compared at high MOI between wild type and knock out conditions.
In general, the authors have a solid observation of the loss of cytoskeletal proteins after RABV infection, but the role of SARM1 might not be limited to the delayed spread (due to delayed axon degeneration). Instead, SARM1 effects might involve innate immunity as well. More detailed characterization of axons vs dendrites is required to make that distinction.

<u>Response:</u> *We acknowledge the reviewer's concern to discriminate between axons and dendrites. However, our data clearly demonstrates the identification and distinction of axons using neurofilament antibody (SMI-312) and dendrites using MAP2 antibody. For reasons stated above and as demonstrated in many previous studies, this staining is highly appropriate and adequate to make the distinction between axons and dendrites. For further clarity, we have now added additional data, showing the identification of axons and dendrites on the same neurons and degeneration of both structures in response to rabies infection (suppl. Fig 2A, results page 6; line 138). Throughout the manuscript, we have described degeneration of both dendrites and axons, not just by the loss of MAP2, but also by the specific loss of neurofilament axonal staining. This is to be expected, as SARM1-mediated axonal degeneration in axonal injury has been shown to induce degeneration of both MAP2 positive-dendrites [13, 14, 15] and neurofilament positive-axons [16, 17, 18]. To inform the readers, this information with citations is now added to the revised manuscript (page 4; line 89). Additionally, we acknowledge that use of the term "axonal degeneration" may not infer both axonal and dendrite degeneration to the readers. In this manuscript, we use the term to describe neurite degeneration, in line with generally used terminology. For clarity, we have now added additional text in the introduction to explain our use of this term (page 4; line 92).*

The loss of both dendrites and axons demonstrated in this study implies that both somadendritic and trans-neuronal spread of rabies could be inhibited by SARM1-mediated mechanism. This is demonstrated in Figure 4, where we have used a specialized microfluidic system. In this model system, we show that trans-neuronal rabies virus transmission is impaired in WT neurons but not in SARM1 knockdown neurons. The viral transmission between the neuronal populations in this model system relies on longer axonal projections for trafficking through microchannels. Hence, while we acknowledge the reviewer's point that the stain does not specifically visualize axons, we believe we are justified in claiming that transmission across the chambers is impaired due specifically to axonal degeneration.

We disagree with the reviewer that this impairment of trans-neuronal spread could simply be due to a role of SARM1 in negative regulation of immune signaling resulting in increased viral replication in knockout neurons. We are able to say this because the spread of rabies virus is measured by a viral titre assay, as shown in Figure 4, where the viral titre ratio between the inoculated and non-inoculated panels is used to account for any such potential difference in viral replication between WT and SARM1 Knockout neurons (Fig 4C, original figure#). We observed no significant difference in virus yield between WT and SARM1 Knockout neurons at 24 hours timepoint, by analyzing the viral titre from the inoculated panel alone. To make the

readers aware of this data, we have added additional graph in Figure 4 to include comparison of individual viral titre values in the inoculated and non-inoculated panels. In this new graph (4C, revised figure#), it can be observed that while there is no significant difference in viral yield in the inoculated panel between WT and SARM1 knockout neurons, it is significantly reduced (~3-fold) in the non-inoculated panel implying an impaired spread of virus.

Therefore, the observed increase in transneuronal spread of rabies virus in SARM1 knockout neurons is not simply due to increased viral replication, but instead due to the delayed loss of axons and dendrites at 24 hours post-infection. We agree with the reviewer that SARM1 has a well-known role in negative regulation of TLR3 and TLR4 mediated immune signaling. However, we did not observe significant increase in viral replication in the SARM1 knockout neurons during or prior to the onset of axonal degeneration in this study. A previous study on West Nile virus has also shown that SARM1 knockout does not influence viral replication in primary neurons [19]. In addition, SARM1 also has a role in activation of neuronal apoptosis in response to viral infection [20]. In this study, we have identified a novel role of SARM1 in anti-viral defense, which appears to be independent of its role in apoptosis and viral replication. However, the interplay between these different functions of SARM1 in anti-viral defense remains to be identified in future studies, particularly at later timepoints. Hence, we have now added the following text to the discussion (page 11; line 265), to describe these additional roles of SARM1.

"This study describes a new anti-viral defense mechanism mediated by SARM1 in response to rabies infection. This adds on to previously established roles of SARM1 in innate immune response such as negative regulation of toll-like receptor (TLR3) signaling [21] and induction of neuronal apoptosis in response to viral infection [20]. SARM1 is also shown to regulate intrinsic neuronal chemokine and cytokine response in traumatic axonal injuries [22]. The functional relationship between these multifaceted roles of SARM1 in innate immunity remains to be investigated."

Reviewer #3: Using rabies virus field isolates, this report shows that, in response to infection, neurons activate selective degeneration of processes, mediated by the loss of NAD and digestion of cytostructural proteins. The authors then show that SARM1, known to be associated with axonal degeneration, is key to this process, likely restricting the ability of the virus to spread trans-synaptically. This is a beautifully done study: the images are compelling, the data are definitive, and the "story" is simple but powerful. Two minor comments:

1. It is difficult to ascertain, even from the high res file, but it appears that the nuclei in infected mice in Figure 1B are smaller and more fragmented than in the mock control. This is also seen in other images presented, but not always consistently. Is this the case? If samples are collected later, do the nuclei remain apparent, or is this a "stepwise" process in which neurites are lost followed by neuronal loss? TUNEL may not capture other forms of cell death.

Response: *It should be noted that Fig 1B shows stitched tile confocal images covering a large area to show the clear loss of MAP2-positive structures. A higher magnification images of nuclei staining in mock-infected and Thai dog rabies infected neurons from the same Figure 1B is shown below. This image shows no significant difference in nuclei fragmentation or condensation between mock-infected and rabies-infected cultures at 24 hours. This is consistent with our data using TUNEL staining. Hence while degeneration of neurites is observed at 24 hours post-infection, the cell bodies remained healthy without any nuclei fragmentation. This shows that neurite degeneration in rabies infection occurs early before the induction of apoptosis.*

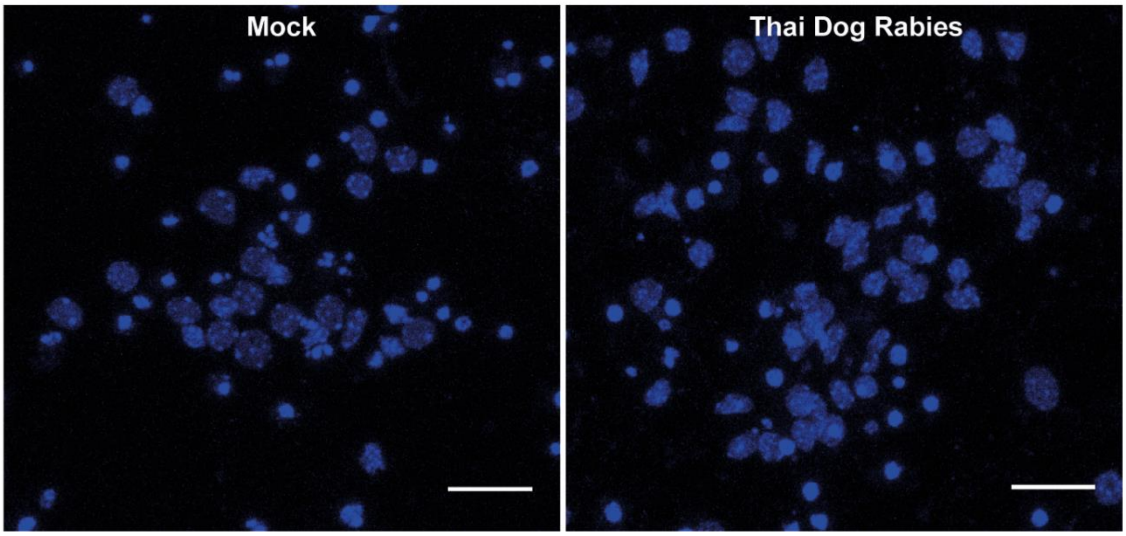

2. This is, exclusively, an ex vivo study using primary neurons. While I do not believe it is compulsory to perform experiments in mice, I am curious if RV-infected, SARM KO mice differ from wild type mice in terms of pathogenesis. Addition of in vivo data (if it is even possible to obtain) would add merit to the significance of the work.

Response: *We agree with the reviewer on the value of studying this novel neurobiological mechanism in animal models. However as stated in the manuscript, this will require well defined wildtype mice models of rabies infection, which consistently and more accurately represent the two clinical forms of rabies. This is essential to conclusively determine the effect of SARM1 knockdown on the clinical pathogenesis of rabies. We are currently working towards establishing such model systems, which however requires a huge volume of work, lying beyond the scope of this manuscript.*

Part II – Major Issues: Key Experiments Required for Acceptance

Reviewer #1: The identification of neuritis as axons is critical. The MAP2 staining is not convince proof that they are imaging axons.

Response: *We are led to believe that the reviewer may have overlooked the existing data in the manuscript showing clear identification of axons by physically separation using microfluidics and specifically staining for axons using SMI-312 antibody. We have now added additional text and changed wording at multiple places in the manuscript to better infer our identification of axons and dendrites. This study shows that both MAP2-postive dendrites and neurofilament-positive axons degenerate in rabies infection, causing an impairment of transneuronal trafficking of rabies virus. We have now added additional data (Suppl. Fig 2A), confirming this mechanism.*

The alternative hypothesis that the effect of SARM1 is to negatively regulate TLR3 and 4 pathways must be considered/discussed.

Response: *The role of SARM1 in negative regulation of TLR3 and neuronal apoptosis is now added to discussion (page 11; line 265).*

Virus yields should be compared at high MOI between wild type and SARM1 knock out infections.

Response: *As stated earlier, there is no significant difference in viral yield between WT and SARM1 knock out neurons after 24 hours infection at MOI of 1. Hence, difference in viral replication could not be the primary mechanism behind the difference in axonal transmission of rabies virus reported in this study.*

Reviewer #2: Although this work is extremely interesting and paves the way for further understanding how RABV can kill its host, I have several concerns.
The choice of the virus strains: The authors claimed they have chosen field strain lyssaviruses in order to limit or avoid any artefact regarding the original pathogenicity. This is a very good statement but, as described in the material and methods, the viruses have been amplified in suckling mouse brain and passaged in Neuro-2a cells (<3 passages). The viruses are then mouse adapted. Therefore, the authors should provide NGS sequencing of the field isolates before, during and after selection and passages in order to show the consensus sequence and the viral population diversity.
On the other hands and on the contrary of the authors, I think a highly virulent laboratory fixed strain, which exhibits Rabies encephalitis in mice, would be extremely informative and should be included in this study.

Response: *We are pleased to see reviewer's agreement to our approach of using field strain lyssavirus to study the original pathogenicity. We also agree with the reviewer that it is essential to eliminate influence on pathogenicity due to possible mouse adaptation during the amplification procedure of our viral strains. Hence, as stated earlier, we have now repeated the experiment with parental field strains before passaging in suckling mouse brain or Neuro-2a cell line. These original field strain infections induced complete degeneration of axons and dendrites at 24 hours post-infection like other viral strains used in this study. Therefore, the minimal adaptation of viral strains has not influenced the ability of these strains to induce axonal degeneration described in this manuscript. We have added this new data in suppl. Fig 2A. Text describing this data is added to the results section (page 6; line 135).*

The use of field isolates of lyssavirus has enabled us to identify a common pathogenic mechanism between different lyssavirus and rabies strains. In the last decades, various fixed laboratory strains of rabies have been established, while some are highly virulent, many vary in their ability to induce specific pathogenic mechanisms. For this reason, we have deliberately avoided using laboratory-adapted or fixed rabies strains in this study. While it will be beneficial to investigate how laboratory fixation affects the virus ability to induce SARM1-mediated axonal degeneration, this additional line of research does not impact the findings of this study. We have described an important pathogenic mechanism common among different field lyssavirus strains which could lead to neurological dysfunction in the host. These findings are likely to be more relevant to natural rabies infection than using laboratory fixed strains.

The choice of the cellular models: All the experiments are undertaken either with primary cortical neurons (embryonic day 15) and primary DRG neurons (embryonic day 13 or 14). Therefore, the study is carried out on immature neurons. Since neuron survival can be neuron type-specific and/or development stage-specific (see for example AJ Kole et al-2013, Ulrich Pfisterer et al-2017), the authors have to establish that their conclusions are still relevant in mature neurons.
Although, I would suggest they show at least some data in iPS derived human neurons in order to extend their findings to other species.

Response: *The neuronal cultures used in this study have mature neurons as evidenced by staining for widely-accepted mature neuronal markers such as MAP2 and neurofilament. However, we do acknowledge that these neurons are developmentally young, and are not aged neurons. Mouse primary neuronal cultures are a well-established model system to study molecular mechanisms of axonal degeneration. This model system has been used to identify several genes associated with axonal self-destruction including SARM1, which have been further validated in adult mouse models [16, 23, 24, 25, 26, 27, 28, 29, 30, 31, 32]. Therefore, we believe that findings from our neuron model in relation to axonal self-destruction are relevant and applicable to mature neurons. Due to practical and technical constraints, it is not feasible to study rabies pathogenesis at a cellular and molecular level directly in aged neurons in living animals. Hence, we have chosen the primary neuronal model system, which has enabled us to identify a key cellular mechanism*

in rabies. Since we have now identified the signaling mechanism at a cellular level, publishing these findings could lead to the investigation of this mechanism in nervous systems using animal models in future studies.

While the iPS or ES derived neural culture system could provide an alternate model to mouse primary neurons and extend these findings in human neurons, this culture system also does not generate aged neurons. In addition, it is not yet clear whether stem cell-derived human neural cultures could develop the complete signaling mechanisms necessary to initiate axonal self-destruction. This needs to be established first before our findings can be extended to stem cell-derived human neurons and lies out of the scope of this study.

Statement: The authors claimed that: "this presents the first identification of a direct mechanism for rabies induced dysfunction of neurons". It looks to me that is a bit overstated. The authors may want to cite other articles such as (not exclusive): Alan C. Jackson et al et al-2010, Jeison Monroy-Gomez et al-2018 and so on.

Response: *While the previous studies have shown axonal and dendritic abnormalities in rabies infection, the key signaling mechanism behind this pathology has been unknown until now. In agreement with the reviewer, we have toned down the statement to one below (page 12; line 304).:*

"To our knowledge, this presents the first identification of the role of SARM1 pathway in evoking axonal degeneration in rabies infection. This describes a novel signaling mechanism responsible for dysfunction of neurons in rabies infection."

We have also cited the two studies indicated by the reviewer in introduction, adding to other studies cited in the original manuscript to describe previously established axonal pathology in rabies (page 5; line 115).

We hope you find our manuscript is now suitable for publication and look forward to hearing from you. We confirm that this work is original and has not been previously published, nor is it currently under consideration for publication elsewhere.

Yours sincerely,

Vinod Sundaramoorthy PhD
Research Scientist-Neurovirology
CSIRO Australian Animal Health Laboratory
vinod.sundaramoorthy@csiro.au
+61 (03) 5227 5210

References

1. Gumy, L.F., *et al.*, *MAP2 defines a pre-axonal filtering zone to regulate KIF1-versus KIF5-dependent cargo transport in sensory neurons.* Neuron, 2017. **94**(2): p. 347-362. e7.
2. Di Stefano, G., *et al.*, *Distribution of map2 in hippocampus and cerebellum of young and old rats by quantitative immunohistochemistry.* Journal of Histochemistry & Cytochemistry, 2001. **49**(8): p. 1065-1066.
3. Yuan, A., *et al.*, *Neurofilaments at a glance*. 2012, The Company of Biologists Ltd.
4. Portier, M., *et al.*, *Peripherin and neurofilaments: expression and role during neural development.* Comptes rendus de l'Academie des sciences. Serie III, Sciences de la vie, 1993. **316**(9): p. 1124-1140.
5. Ulfig, N., *et al.*, *Monoclonal antibodies SMI 311 and SMI 312 as tools to investigate the maturation of nerve cells and axonal patterns in human fetal brain.* Cell and tissue research, 1998. **291**(3): p. 433-443.
6. Haynes, R.L., *et al.*, *Axonal development in the cerebral white matter of the human fetus and infant.* Journal of Comparative Neurology, 2005. **484**(2): p. 156-167.
7. Christensen, K.R., *et al.*, *Pathogenic tau modifications occur in axons before the somatodendritic compartment in mossy fiber and Schaffer collateral pathways.* Acta neuropathologica communications, 2019. **7**(1): p. 29.
8. Bony, G., *et al.*, *Non-hyperpolarizing GABA B receptor activation regulates neuronal migration and neurite growth and specification by cAMP/LKB1.* Nature communications, 2013. **4**: p. 1800.
9. Beffert, U., *et al.*, *Microtubule plus-end tracking protein CLASP2 regulates neuronal polarity and synaptic function.* Journal of Neuroscience, 2012. **32**(40): p. 13906-13916.
10. Pongrakhananon, V., *et al.*, *CAMSAP3 maintains neuronal polarity through regulation of microtubule stability.* Proceedings of the National Academy of Sciences, 2018. **115**(39): p. 9750-9755.
11. Chander, P., *et al.*, *Neuron-Specific Gene 2 (NSG2) encodes an AMPA receptor interacting protein that modulates excitatory neurotransmission.* eNeuro, 2019. **6**(1).
12. Taylor, A.M., *et al.*, *A microfluidic culture platform for CNS axonal injury, regeneration and transport.* Nature methods, 2005. **2**(8): p. 599.
13. Folkerts, M.M., *et al.*, *Disruption of MAP-2 immunostaining in rat hippocampus after traumatic brain injury.* Journal of neurotrauma, 1998. **15**(5): p. 349-363.
14. Taft, W.C., *et al.*, *Hypothermia attenuates the loss of hippocampal microtubule-associated protein 2 (MAP2) following traumatic brain injury.* Journal of Cerebral Blood Flow & Metabolism, 1993. **13**(5): p. 796-802.
15. Gao, X., *et al.*, *Moderate traumatic brain injury causes acute dendritic and synaptic degeneration in the hippocampal dentate gyrus.* PloS one, 2011. **6**(9): p. e24566.
16. Geisler, S., *et al.*, *Gene therapy targeting SARM1 blocks pathological axon degeneration in mice.* Journal of Experimental Medicine, 2019. **216**(2): p. 294-303.
17. Marion, C.M., *et al.*, *Sarm1 deletion reduces axon damage, demyelination, and white matter atrophy after experimental traumatic brain injury.* Experimental neurology, 2019. **321**: p. 113040.
18. Yang, J., *et al.*, *Regulation of axon degeneration after injury and in development by the endogenous calpain inhibitor calpastatin.* Neuron, 2013. **80**(5): p. 1175-1189.
19. Szretter, K.J., *et al.*, *The immune adaptor molecule SARM modulates tumor necrosis factor alpha production and microglia activation in the brainstem and restricts West Nile Virus pathogenesis.* Journal of virology, 2009. **83**(18): p. 9329-9338.
20. Mukherjee, P., *et al.*, *Activation of the innate signaling molecule MAVS by bunyavirus infection upregulates the adaptor protein SARM1, leading to neuronal death.* Immunity, 2013. **38**(4): p. 705-716.
21. Carty, M., *et al.*, *The human adaptor SARM negatively regulates adaptor protein TRIF–dependent Toll-like receptor signaling.* Nature immunology, 2006. **7**(10): p. 1074.
22. Wang, Q., *et al.*, *Sarm1/Myd88-5 regulates neuronal intrinsic immune response to traumatic axonal injuries.* Cell reports, 2018. **23**(3): p. 716-724.

23. Gilley, J., *et al.*, *Absence of SARM1 rescues development and survival of NMNAT2-deficient axons.* Cell reports, 2015. **10**(12): p. 1974-1981.
24. Summers, D.W., *et al.*, *SARM1-specific motifs in the TIR domain enable NAD+ loss and regulate injury-induced SARM1 activation.* Proceedings of the National Academy of Sciences, 2016. **113**(41): p. E6271-E6280.
25. Geisler, S., *et al.*, *Vincristine and bortezomib use distinct upstream mechanisms to activate a common SARM1-dependent axon degeneration program.* JCI insight, 2019. **4**(17).
26. Sasaki, Y., *et al.*, *NMNAT1 inhibits axon degeneration via blockade of SARM1-mediated NAD+ depletion.* Elife, 2016. **5**: p. e19749.
27. Osterloh, J.M., *et al.*, *dSarm/Sarm1 is required for activation of an injury-induced axon death pathway.* Science, 2012. **337**(6093): p. 481-484.
28. Gilley, J., *et al.*, *Sarm1 deletion, but not WldS, confers lifelong rescue in a mouse model of severe axonopathy.* Cell reports, 2017. **21**(1): p. 10-16.
29. Henninger, N., *et al.*, *Attenuated traumatic axonal injury and improved functional outcome after traumatic brain injury in mice lacking Sarm1.* Brain, 2016. **139**(4): p. 1094-1105.
30. Geisler, S., *et al.*, *Prevention of vincristine-induced peripheral neuropathy by genetic deletion of SARM1 in mice.* Brain, 2016. **139**(12): p. 3092-3108.
31. Walker, L.J., *et al.*, *MAPK signaling promotes axonal degeneration by speeding the turnover of the axonal maintenance factor NMNAT2.* Elife, 2017. **6**: p. e22540.
32. Sasaki, Y., *et al.*, *Transgenic mice expressing the Nmnat1 protein manifest robust delay in axonal degeneration in vivo.* Journal of Neuroscience, 2009. **29**(20): p. 6526-6534.
